# SIGNIFICANT DIFFERENCES IN EVALUATION OF DISABILITY AND HEALTH (ICF) CORE SETS BETWEEN STROKE REHABILITANTS AND REHABILITATION TEAM DURING THE FIRST YEAR

**DOI:** 10.2340/jrm.v58.42856

**Published:** 2026-02-06

**Authors:** Aet RISTMÄGI, Hannu HEIKKILÄ, Olavi AIRAKSINEN

**Affiliations:** From the Department of physical medicine and rehabilitation, Kuopio University hospital, Finland

**Keywords:** stroke, rehabilitation, health-related quality of life, ICF, disability, functioning

## Abstract

**Objective:**

Assessing functional abilities in stroke rehabilitation is essential, combining subjective self-reports with objective clinical evaluations.

**Methods:**

This study aimed to compare self- reported impairments from stroke patients with rehabilitation team evaluations using the ICF stroke core set at 3 time points: 1 month post-discharge, after 6 months, and 12 months post-diagnosis. Additionally, the study sought to identify ICF subdomains most impacting health-related quality of life (HRQOL) as measured by EQ-5D. This longitudinal, retrospective observational study included consecutive 118 stroke patients at the Satahospital Rehabilitation Unit (2021–2022).

**Results:**

Results showed that, 1 month after discharge, patients rated their functioning higher than team assessments, particularly in cognitive domains. By 12 months, patients’ self-reports indicated lower functioning than team evaluations, with discrepancies diminishing over time. Objective assessments revealed significant improvements in mobility, self-care, and cognitive functions, while patients reported progress in life activities and social interactions but little change in physical or cognitive domains. Depression levels and self-care ability (washing) were the strongest predictors of improved HRQOL.

**Conclusion:**

These findings reveal that patients initially overestimate their abilities, influenced by a lack of awareness and emotional factors, while rehabilitation teams provide more objective evaluations and individualized rehabilitation. Integrated assessment frameworks combining subjective and objective perspectives are crucial to optimizing rehabilitation outcomes.

## LAY ABSTRACT

-The first-year rehabilitation outcomes of stroke patients reveal significant differences in functioning between self-assessments by stroke survivors and evaluations by the rehabilitation team but the overall disagreement diminished at the 12-month follow-up.
-Objective assessments indicated significant improvements in cognitive, motor, and ADL skills, while patients reported greater enhancements in work activity, household tasks, community participation
-Cognitive domains exhibited the greatest inter-observer differences, with patients perceiving restrictions as less severe.
-The strongest predictors of quality of life, as measur-ed by the EQ-5D, were self-washing and depression.
- The ICF core set, encompassing both objective and subjective assessments from patients and rehabilitation teams, proves to be a valuable instrument for measuring rehabilitation outcomes.

These insights underscore the importance of integrating both patient-reported outcomes and clinical evaluations to effectively address the multifaceted nature of stroke recovery.

Late subacute stroke rehabilitation presents challenges, as functional improvement tends to decrease after 3 months post-diagnosis. By 6 months, no significant alterations in physical functioning are typically expected (1). Emphasis should be placed on strategies that enhance overall quality of life and empower individuals to navigate their environments and social contexts more effectively (2, 3).

A lack of awareness, or anosognosia, is a common post-stroke complication that significantly hinders rehabilitation outcomes. This deficit can involve unawareness of motor, sensory, or cognitive impairments, such as paralysis, and negatively impacts adherence to therapy and functional recovery, as patients may underestimate their limitations or not engage in treatment as effectively (4–7). A limited number of studies deal with coping as part of the psychological adaptation process after stroke (8). However, the coping strategies that people use after a stroke may influence recovery (8). Coping is the general plan we have at our disposal for dealing with stress (9). Coping is proposed to be critical for understanding patients’ behaviour and serves as an important part of any treatment to be offered (9, 10). Graven et al. (11) conducted a systematic review of the relationships between coping and health-related outcomes for cardiac patients. Effective coping involves recognizing the validity of feelings like frustration and anxiety. Problem-focused coping was generally associated with improved psychological well-being, self-care, and health-related quality of life, while emotion-focused coping was associated with poorer psychological well-being and health-related quality of life, and a higher risk of mortality. Furthermore, emotion-focused coping was associated with adverse outcomes such as increased anxiety, emotional exhaustion, and dissatisfaction, while problem-focused coping was associated with lower levels of emotional exhaustion. When used in a maladaptive way, such as with denial, avoidance, or toxic positivity, emotion-focused coping is not helpful.

The International Classification of Functioning (ICF) is a widely used tool for assessing post-stroke functioning and is valuable for rehabilitation goal setting (12). ICF has stroke-specific core sets, primarily designed for trained rehabilitation specialists, but self-administered patient versions of ICF, such as the WHODAS 2.0, are also in use. ICF core sets have been extensively researched, with their validity and reliability fully supported (13–15). Some longitudinal stroke studies have demonstrated the utility of ICF in follow-up care. Additionally, there are studies comparing the perceptions of functioning between stroke patients, caregivers, and healthcare professionals using ICF (16–19). Previous studies have reported discrepancies in perceived disability, with patients rating their restrictions as less severe compared with caregivers or rehabilitation specialists. Discrepancies between subjective and objective assessments of functional abilities can significantly impact HRQOL, goals, and outcomes in stroke rehabilitation. Integrating both types of assessments and addressing psychological factors may improve rehabilitation effectiveness and patient satisfaction.

The aim of this longitudinal retrospective registry study is to compare perceived functional restrictions between rehabilitants and multidisciplinary rehabilitation teams at the 1-year follow-up using the ICF stroke core set. The second aim is to explore the correlation between ICF subdomains and quality of life, as measured by the EQ-5D, during the first year after stroke.

## MATERIALS AND METHODS

### Study design and participants

Participants in this study were 118 consecutive people who had an ischaemic or haemorrhagic stroke between 18 January and 17 December 2022, and participated in rehabilitation at the multidisciplinary rehabilitation unit at Satahospital (SCH) in Finland. The study is a longitudinal retrospective single-centre registry study. A multidisciplinary rehabilitation team followed up all patients at least 1 year after their stroke diagnosis. We assessed the ICF core set and HRQOL 3 times during the follow-up: once 1 month after the rehabilitation unit discharge, once at 6 months, and once at the 12-month follow-up after diagnosis. Informed consent was obtained from all individuals included in this study. Data collection was carried out according to European data protection law. The data were collected from the Quality Registry Database of the unit (THL/2182/5.09.00/2019). Research involving human subjects complied with all relevant national regulations and institutional policies and is in accordance with the tenets of the Helsinki Declaration (as amended in 2013). The Regional Ethics Committee, Satakunnan Sairaanhoitopiiri (SATSHP/697/13.01/2020), granted ethical approval.

### Outcome assessment

The Satahospital (SCH) rehabilitation unit elaborates on the ICF core set, including 37 subdivisions, also to encompass the sub-sections of FIM (Functional Independence Measure) (20), Finnish HRQOL Questionnaire 15D (21), and WHODAS 2.0 short version items (World Health Organization Disability Assessment Scale) (22).

We assessed the ICF core set 3 times during the follow-up: once 1 month after the rehabilitation unit discharge, once at 6 months, and once at the 12-month follow-up after diagnosis. The ICF core set in our unit is used for assessing disability and for patient-centred goal-setting: once 1 month after the rehabilitation unit discharge, once at 6 months, and once at the 12-month follow-up after diagnosis.

Rehabilitants and a multidisciplinary team conduct separate assessments for 30 subdivisions, both blinded by opinions from other parties. For 7 subdivisions (sleeping, pain, sexual function, housework, work activity, joining in community activities, and maintaining friendship), we have patients’ assessment only. The rehabilitation team, which included a doctor of physical medicine and rehabilitation, a clinical psychologist, a physiotherapist, a stroke nurse, an upper limb rehabilitation specialist, and a rehabilitation instructor, received training in the ICF coding structure and had written instructions for assessment. Meanwhile, the patient received general instructions based on the ICF manual. All patients had the same instructions from the same rehabilitation instructor at any follow-up appointment. If the patient declined to participate in rehabilitation but consented to follow-up, the stroke nurse made phone contact, documented only the 12-item WHODAS 2.0, and we conducted no team assessment.

We used WHODAS 2.0 short version as part of the ICF assessment. According to the ICF, the WHODAS 2.0 measures a person’s level of functioning in 6 main areas of their life: cognition (understanding and communication), mobility (ability to move and get around), self-care (ability to take care of personal hygiene, dress, and eat alone), getting along (ability to get along with others), life activities (ability to carry out responsibilities at home, work, and school), and participation in society (ability to engage in community, civil, and recreational activities). The short version of WHODAS 2.0 explains 81% of the variance in the 36-item version. For each domain, the 12-item version includes 2 sentinel items with good screening properties that identify over 90% of all individuals with even mild disabilities when tested on all 36 items (23). We used 6 WHODAS 2.0 subdomains (cognition, mobility, self-care, participation, life activity, getting along) to identify the areas with differences or improvements. ICF assessment including WHODAS 2.0 items was achieved from 95 patients at 1 month control, and for 6- and 12-month follow-up. 17 patients were unable to complete any form of ICF due to insufficient linguistic or cognitive skills, or they chose not to respond due to their overall weak health condition. ICF scores are originally categorized into 5 grades: no problem (0–4%), mild problem (5–24%), moderate disability problem (25–49%), severe problem (50–95%), and extreme problem (96–100%). As for patient self-assessment, patients answered the question as in the usual Finnish version of ICF questionnaire, with “how much of a problem do you have...” with possible answers “none”, “mild”, “moderate”, „severe”, “extreme or cannot do”. For ICF, we changed the original point-calculation results to index from 0 to 1, with 0 indicating worst performance and 1 indicating best performance. We calculated “none” as 1.0, “mild” as 0.75, “moderate” as 0.50, “severe” as 0.25, and “extreme or cannot do” as 0. According to the calculation by SPSS, all patients had an ICF index from 0 to 1.

The European HRQOL questionnaire EQ-5D comprises 5 questions on mobility, self-care, pain, usual activities, and psychological status and has 3 possible answers for each item, namely 1 = no problem, 2 = moderate problem, 3 = severe problem (24, 25). A summary index with a maximum score of 1 can be derived from these 5 dimensions by conversion with a table of scores. The maximum score of 1 indicates the best health state. In contrast, high scores on the individual questions indicate more severe or frequent problems. It also has a visual analogue scale (VAS) to indicate general health status, with 100 indicating the best health status (26). Health-related quality of life scores, which usually range from 0 (death) to 1 (full health), are frequently used to assign value to the level of HRQOL and are shown to be in the range of 0.45 to 0.59 according to the Swedish national stroke rehabilitation register in acute and early subacute phases (https://svereh.registercentrum.se).

### Statistical analysis

All statistical analyses were carried out in the IBM Statistical Package for Social Sciences (IBM SPSS Statistics 28.0.0.0; IBM Corp, Armonk, NY, USA). We used the Wilcoxon signed-rank test for two dependent samples, or the Kruskal-Wallis test for independent samples to compare magnitude and direction of difference between distributions of scores, to compare the patient’s subjective opinion to the rehabilitation team’s objective assessment. We stated the significance level at a *p*-value less than 0.05. We studied the ICF core set subdomains and their relation to EQ-5D using correlation analysis. Summary variables of the different aspects of functioning were computed to assess their association using Spearman’s rho coefficients (rs), with particular care being taken to ensure that the computed variables were indeed comparable. The agreement coefficients were interpreted as *r* < 0.1, Very small; 0.1 < = *r* < 0.3, Small; 0.3 < = *r* < 0.5, Moderate; *r* > = 0.5, Large (27). Only correlations above 0.4 were reported as significant in this study. We used the stepwise linear regression model to find the best ICF subdomains to predict HRQOL, EQ-5D at 1-year follow-up.

### Data availability statement

The data associated with the paper are not publicly available, but can be obtained from the corresponding author on reasonable request.

## RESULTS

General characteristics are listed in Table I.

**Table I:** General characteristics of patients attending post-stroke rehabilitation in Satahospital (SCH), Department of Physical Medicine and Rehabilitation

Variable	*n* (%)
Sex (male)	64 (54)
Age, years, mean	66.7
Min	20
Max	91
Diagnosis	
Ischaemic stroke	94 (80)
Haemorrhagic stroke	24 (20)
Patients attended multidisciplinary rehabilitation	118
Outpatient rehabilitation only	13
Hospital Rehabilitation Unit (SCH)	105
Discharge from hospital	118
Independent	62 (53)
With assistance	56 (47)
Discharge from SCH	105
Home	83 (79)
Nursing home	6 (6)
Other rehabilitation/general ward	16 (15)
12-months’ follow-up	
New vascular event	6 (5)
Other complication	4 (3)
Alive	116 (98)
Living at home	111 (94)
Independent	84 (76)
With assistance	27 (24)
Living in nursing home	5 (4)

### Assessment at 1 month after discharge

At 1 month after discharge, patients consistently estimated their restrictions in various subdomains as less severe compared with the assessments provided by the rehabilitation team (Table II). Patients rated their performance significantly higher in cognitive domains such as concentration, learning new tasks, memory functions, and problem-solving, as well as in emotional functions, including depression and anxiety. Additionally, patients reported significantly better performance in certain functional domains related to self-care and mobility, such as washing, dressing, toileting, transferring to the toilet and bathroom, walking, and walking on stairs, compared with the rehabilitation team’s assessments. However, the differences were most pronounced in cognitive domains. At 1 month after discharge, there was no area where patients reported inferior outcomes compared with the team’s assessments.

**Table II:** Significant differences in ICF subdomain assessments at first control after discharge

Item	Mean	SD	Standard error	Z	Asymp.	Sign 2-sided
Concentration, Patient	0.91	0.18	86.8	–3.20		0.001
Concentration, Team	0.85	0.19				
Learning new tasks, Patient	0.88	0.2	75.9	–3.24		0.001
Learning new tasks, Team	0.73	0.2				
Memory, Patient	0.84	0.2	83.3	–3.40		< 0.001
Memory, Team	0.75	0.17				
Emotional affection, Patient	0.83	0.2	62.1	–3.36		< 0.001
Emotional affection, Team	0.72	0.21				
Anxiety, Patient	0.85	0.22	63.8	–3.43		< 0.001
Anxiety, Team	0.78	0.22				
Solving problems, Patient	0.87	0.2	62.3	–2.57		0.01
Solving problems, Team	0.80	0.2				
Depression, Patient	0.85	0.22	49.5	–2.33	0.08	0.02
Depression, Team	0.80	0.21				
Washing, Patient	0.94	0.16	50.1	–4.0		< 0.001
Washing, Team	0.83	0.22				
Dressing, Patient	0.95	0.15	35.5	–3.52		< 0.001
Dressing, Team	0.88	0.18				
Toileting, Patient	0.98	0.08	10.3	–2.67		0.008
Toileting, Team	0.95	0.12				
Walking on stairs, Patient	0.84	0.22	48.7	–3.13		0.002
Walking on stairs, Team	0.76	0.27				
Transfer to shower, Patient	0.95	0.14	20.6	–3.71		< 0.001
Transfer to shower, Team	0.89	0.17				
Transfer to toilet, Patient	0.97	0.1	17.4	–2.98		0.003
Transfer to toilet, Team	0.92	0.15				
Walking, Patient	0.81	0.31	58.0	–2.01		0.04
Walking, Team	0.78	0.23				

The table outlines the significant differences in International Classification of Functioning, Disability, and Health (ICF) subdomains between the patients’ self-assessments and the rehabilitation team’s assessments. Values range from 0 (worst function) to 1 (no problems), indicating perceived restrictions in functioning.

### Assessment at 6 months’ follow-up

Compared with the first assessment, agreement between the rehabilitation team and patient assessments improved in most for fine motor skills of the hand and cognitive domains. However, discrepancies persisted in certain functional areas, with patients reporting inferior performance compared with team evaluations. At the 6-month follow-up, patients continued to perceive better performance in cognitive domains compared with the rehabilitation team’s assessments (Table III). The most notable difference was observed in emotional aspects, such as depression and anxiety. In motor skills, patients still reported superior performance compared with the team’s assessment in activities such as washing, dressing, and transferring to the toilet. Additionally, patients perceived grooming (caring for body parts) as less difficult than the team’s evaluation. However, at the 6-month follow-up, patients perceived urination and defecation functions, as well as fine hand use, as more problematic than the rehabilitation team’s assessment.

**Table III:** Significant differences in ICF subdomain assessments at 6-month follow-up

Item	Mean		SD	Standard error	Z	Asymp.	Sign 2-sided
Anxiety, Patient	0.88		0.16	39.5	–3.43		< 0.001
Anxiety, Team	0.81		0.19				
Emotional affection, Patient	0.85		0.2	56.7	–2.27		0.02
Emotional affection, Team	0.74		0.17				
Depression, Patient	0.88		0.1	43,7	–2,67		0.008
Depression, Team	0.81		0.18				
Washing, Patient	0.93		0.19	30.3	–3.14		0.002
Washing, Team	0.82		0.22				
Dressing, Patient	0.94		0.15	34.0	–3.08		0.002
Dressing, Team	0.85		0.2				
Caring for body parts, Patient	0.91		0.16	25.7	–2.45		0.01
Caring for body parts, Team	0.87		0.15				
Transfer to toilet, Patient	0.95		0.16	21,0	–2.02		0.04
Transfer to toilet, Team	0.92		0.15				
Defecation, Patient	0.94		0.15	20.6	3.27		0.001
Defecation, Team	0.97		0.09				
Urination, Patient	0.91		0.18	26.9	2.84		0.005
Urination, Team	0.92	75	0.1				
Fine motor hand use, Patient	0.71		0.29	53.2	3.63		< 0.001
Fine motor hand use, Team	0.81		0.22				

The table illustrates significant differences in International Classification of Functioning, Disability, and Health (ICF) subdomains at 6-month follow-up. Values range from 0 (worst function) to 1 (no problems).

### Assessment at 12 months’ follow-up

While the overall disagreement between patient and team assessments has diminished, patients tended to rate their functioning as lower in some areas. However, patients consistently perceived inferior functioning in domains such as fine motor hand use, urination, defecation, and transferring to the toilet (Table IV). At the 12-month follow-up, patients still perceived better performance in depression and washing oneself compared with the rehabilitation team’s assessment. However, patients reported significantly worse functioning in listening, urinating, defecation, and fine hand use. There was also a trend for patients to rate their performance as inferior in both cognitive and motor subdomains at the 12-month follow-up, although this was not statistically significant. Compared with the first assessment, there was less disagreement between patients and the rehabilitation team regarding the performance of activities.

**Table IV:** Significant differences in ICF subdomain assessments at 1-year follow-up

Item	Mean	SD	Standard error	Z	Asymp.	Sign 2-sided
Depression, Patient	0.84	0.21	32.5	–2.31		0.02
Depression, Team	0.79	0.19				
Washing, Patient	0.94	0.16	28.4	–2.30		0.02
Washing, Team	0.83	0.23				
Defecation, Patient	0.96	0.11	28.4	2.66		0.008
Defecation, Team	0.94	0.14				
Urination, Patient	0.91	0.18	31.3	1.98		0.05
Urination, Team	0.88	0.2				
Listening, Patient	0.91	0.2	18.4	2.64		0.008
Listening, Team	0.97	0.09				
Fine motor hand use, Patient	0.67	0.29	63.1	3.45		< 0.001
Fine motor hand use, Team	0.79	0.24				

The table highlights significant differences in International Classification of Functioning, Disability, and Health (ICF) subdomains between patient and rehabilitation team assessments at the 1-year follow-up. Values range from 0 (worst function) to 1 (no problems).

### Patients’ experience of performance in longitudinal perspective compared with team assessment

Differences between patients’ subjective ratings and the rehabilitation team’s assessments were notable in several aspects of functioning. One year after the stroke, significant subjective improvements were reported in work activity (Z = 3.41, *p* > 0.001), improvement of concentration (Z = 2.48, *p* = 0.01), improvement in lifting and carrying objects (Z = 2.29, *p* = 0.02) and improvement in learning new tasks (Z = 2.17, *p* = 0.03).

Significant negative changes in experienced impairment and disability were observed in distress 0.80 vs 0,73 (Z = –2.48, *p* = 0.013) and tendency to impaired urination function 0.92 vs 0.90 (Z = –1.80, *p* = 0.07).

In the first 6 months, patients reported improvements in work activity, household tasks, participation in community activities, lifting and carrying objects, and memory functions (Table V). The only significant change between the 6- and 12-month follow-ups was an increase in anxiety from 0.87 to 0.81 (Z = –2.22 *p* = 0.03), decrease in walking ability from 0.82 to 0.79 (Z = –2.64, *p* = 0.008), and improvement in concentration from 0.92 to 0.94 (Z = 2.67, *p* = 0.008).

**Table V:** Subjective reported significant changes in ICF subdomains between 1-month control, 6-month follow-up, and 12-month follow-up

Item	Mean	SD	Standard error	Z	Asymp.	Sign 2-sided
Work activity 1 month	0.84	0.29	267.5	3.63		< 0.001
Work activity 6 months	0.87	0.25				
Lifting objects 1 month	0.73	0.29	40.9	3.11		0.002
Lifting objects 6 months	0.73	0.28				
Emotional affection 1 month	0.83	0.2	471	2.84		0.005
Emotional affection 6 months	0.85	0.2				
Memory 1 month	0.84	0.2	19	2.24		0.02
Memory 6 months	0.86	0.17				
Work activity 1 month	0.84	0.29	336	3.4		< 0.001
Work activity 12 months	0.88	0.24				
Distress 1 month	0.80	0.22	44.7	–2.48		0.01
Distress 12 months	0.73	0.26				
Concentration 1 month	0.91	0.18	227	2.48		0.01
Concentration 12 months	0.94	0.14				
Lifting objects 1 month	0.73	0.29	42	2.29		0.02
Lifting objects 12 months	0.71	0.33				
Learning new tasks 1 month	0.88	0.2	314	2.17		0.03
Learning new tasks 12 months	0.90	0.9				

This table highlights significant improvements (*p* < 0.05) in 4 (ICF) subdomains at 1–6 months’ follow-up and 5subdomains at 1–12 months’ follow-up, as assessed by patients. Values range from 0 (worst function) to 1 (no problems).

According to the rehabilitation team’s assessments at 1 month after discharge and at the 1-year follow-up, there was statistically significant improvement in 18 subdomains (Table VI). No significant changes were observed in excretion functions (urination and defecation), vision, communication (speaking and general understanding), emotional affection caused by health problems, depression, anxiety, energy level, breathing, toileting, or transferring to a chair.

**Table VI:** Significant improvements in ICF subdomains between 1-month control and 12-month follow-up for team assessments

Item	Mean	SD	Standard error	Z	Asymp.	Sign 2-sided
Memory 1 month	0.75	0.17	33.6	4.48		< 0.001
Memory 12 months	0.86	0.16				
Solving problems 1 month	0.80	0.2	33.1	4.16		< 0.001
Solving problems 12 months	0.89	0.16				
Concentration 1 month	0.75	0.19	25.7	3.67		< 0.001
Concentration 12 months	0.83	0.19				
Learning new tasks 1 month	0.73	0.21	26.2	3.27		0.001
Learning new tasks 12 months	0.81	0.19				
Interaction 1 month	0.86	0.22	17.4	2.89		0.003
Interaction 12 months	0.91	0.18				
Washing 1 month	0.83	0.22	15.9	3.3		< 0.001
Washing 12 months	0.83	0.2				
Dressing upper body 1 month	0.87	0.18	14,0	3.21		0.001
Dressing upper body 12 months	0.90	0.2				
Dressing lower body 1 month	0.88	0.18	17	3.0		0.003
Dressing lower body 12 months	0.94	0.2				
Eating 1 month	0.87	0.16	13	3.0		0.003
Eating 12 months	0.93	0.17				
Grooming 1 month	0.89	0.16	12.6	2.50		0.01
Grooming 12 months	0.93	0.16				
Transfer to shower 1 month	0.89	0.17	7.5	3.0		0.003
Transfer to shower 12 months	0.93	0.22				
Walking 1 month	0.76	0.24	30.2	2.83		0.005
Walking 12 months	0.84	0.25				
Lifting objects 1 month	0.74	0.26	24.8	2.42		0.018
Lifting objects 12 months	0.80	0.28				
Fine motor hand use 1 month	0.80	0.22	18.5	2,67		0.008
Fine motor hand use 12 months	0.86	0.24				
Walking on stairs 1 month	0.75	0.27	24.0	2.29		0.02
Walking on stairs 12 months	0.81	0.82				
Transfer to toilet 1 month	0.92	0.15	6.63	2.11		0.03
Transfer to toilet 12 months	0.95	0.19				
Standing 1 month	0.82	0.2	25.7	2.04		0.04
Standing 12 months	0.87	0.22				
Listening 1 month	0.94	0.15	3.6	2.06		0.04
Listening 12 months	0.98	0.1				

This table highlights significant improvements in 18 International Classification of Functioning, Disability, and Health (ICF) subdomains as assessed by the rehabilitation team. Values range from 0 (worst function) to 1 (no problems). The improvements reflect changes in team assessments of patient functioning between the 1-month control and the 12-month follow-up.

### Changes in WHODAS 2.0 subdomains

The ICF core set included WHODAS 2.0 short version, and the rehabilitation team assessed 3 subdomains: cognition (concentration and learning new tasks), mobility (standing and walking), and self-care (washing and dressing oneself). Statistically significant improvements were identified in all subdomains during the first year. For cognition, the mean was 0.75 vs 0,84 (Z = 3.76, *p* < 0.001); for mobility, the mean was 0.79 vs 0.86 (Z = 2.78, *p* = 0.005); and for self-care, the mean difference was 0.86 vs 0.92 (Z = 3.34. *p* < 0.001).

Based on the patients’ subjective evaluations, there were no statistically significant differences in mobility, 0.83 vs 0.82 (Z = –0.92, *p* = 0.35), cognition 0.92 vs 0.90, (Z = –0.85, *p* = 0.39), self-care 0.95 vs 0.95 (Z = 0.87, *p* = 0.38), participation 0.86 vs 0.97 (Z = 0.65, *p* = 0.51), and getting along 0.95 vs 0.96 (Z = 1.89, *p* = 0.059).

However, patients reported significant improvement in life activities 0.85 vs 0.88 (Z = 2.62, *p* = 0.009).

### Regression analysis for health-related quality of life at 1-year follow-up

The best ICF subdomains to predict EQ-5D at 12 months were washing and depression (R square 0.525, *p* < 0.001), with B-slope for washing being 0.54 (95% CI 0.36–0.72, *p* < 0.001) and 0.354 (95% CI 0.17–0.54, *p* < 0.001) for depression and the constant being –0.165.

### Correlations between EQ-5D and ICF core set subdomains at 1-month and 12-month follow-up

At 1-month follow-up, washing and dressing showed substantial correlation with EQ-5D (Table VII). Many of the motor and ADL functions were moderately correlated with EQ-5D. At 1-month control, there was no significant relationship between cognitive skills and EQ-5D. However, we observed a moderate correlation with EQ-5D and many cognitive skill domains at 12-month control.

**Table VII:** Patient-reported outcome

Item	EQ-5D 1-month	EQ-5D 12-month
Household tasks	0.59	0.73
Walking	0.50	0.68
Lifting and carrying objects	0.47	0.66
Washing	0.64	0.64
Dressing upper body	0.63	0.62
Sexual functions	NS	0.61
Work activity	0.51	0.61
Depression	0.42	0.59
Walking stairs	0.50	0.59
Standing	0.56	0.58
Grooming	0.41	0.57
Learning new tasks	NS	0.54
Solving problems	NS	0.53
Urination	NS	0.51
Dressing lower body	0.63	0.51
Emotional affection	0.40	0.49
Joining in community activity	0.50	0.49
Anxiety	NS	0.47
Defecation	NS	0.47
Transfer to shower/bath	0.45	0.46
Fine hand use	0.51	0.46
Concentration	NS	0.45
Toileting	NS	0.44
Eating	NS	0.44
Transfer to chair	0.43	0.43
Energy level	0.42	0.42
Maintaining friendship	NS	0.42
Interaction	NS	0.42
Seeing	0.45	NS
Pain	0.43	NS
WHODAS life activity	0.59	0.71
WHODAS mobility	0.57	0.68
WHODAS self-care	0.64	0.67
WHODAS participation	0.55	0.63
WHODAS cognition	NS	0.56
WHODAS getting along	NS	0.46

This table presents Spearman’s rho coefficients (*r*s) measuring the correlation between ICF core set subdomains and EQ-5D health-related quality of life scores at 1-month and 12-month follow-ups. Interpretation of correlation strength: *r* < 0.1, Very small; 0.1 < = *r* < 0.3, Small; 0.3 < = *r* < 0.5, Moderate; *r* > = 0.5, Large.

### Correlation between WHODAS 2.0 six subdomains and depression

ICF subdomains correlated relatively well with each other. Cognitive skills, getting along, and participation exhibited moderate to substantial correlations with each other at all time points (Table VIII). Similarly, mobility, ADL (activities of daily living), and life activities showed moderate correlations with each other at all time points.

**Table VIII:** Correlations between 6 WHODAS 2.0 subdomains at the 1-month control and 6 subdomains at 12-month control

Item	Mobility	ADL	Getting along	Life activity	Participation
1-month control					
Cognitive skills	NS	NS	0.57	0.59	0.60
Mobility	–	**0.69**	NS	**0.62**	NS
ADL	**0.69**	–	NS	**0.63**	0.48
Getting along	NS	NS	–	NS	**0.63**
Life activity	**0.62**	**0.63**	NS	–	0.55
Participation	NS	0.48	**0.63**	0.55	–
12-month control					
Cognitive skills	0.55	0.45	0.57	0.53	**0.62**
Mobility	–	**0.64**	0.46	**0.76**	**0.62**
ADL	**0.64**	–	0.47	**0.69**	0.51
Getting along	0.46	0.47	–	0.52	0.59
Life activity	**0.76**	**0.69**	0.52	–	**0.62**
Participation	**0.62**	0.51	0.59	**0.62**	–

Spearman’s rho coefficients (ρ) were used to determine the strength of the relationships and are interpreted as follows *r* < 0.1, Very small; 0.1 < = *r* < 0.3, Small; 0.3 < = *r* < 0.5, Moderate; *r* > = 0.5, Large. Substantial correlations are indicated in bold

Depression was correlated with getting along and participation at each follow-up assessment (Table IX). Compared with the 1-month follow-up, the correlation between ICF subdomains improved at the 12-month follow-up, with almost all subdomains showing moderate to substantial correlations at the 12-month follow-up. However, at the 12-month follow-up, depression, mobility, and ADL showed no correlation.

**Table IX:** Correlations between WHODAS 2.0 6 subdomains and ICF depression at 1-month control and 12-month control

Item	Depression, 1 month	Depression, 12 months
Cognitive skills	NS	0.51
Mobility	NS	NS
ADL	NS	NS
Getting along	0.49	0.54
Life activity	NS	0.46
Participation	0.55	**0.64**

Spearman’s rho coefficients (*r*s), were interpreted *r* < 0.1, Very small; 0.1 < = *r* < 0.3, Small; 0.3 < = *r* < 0.5, Moderate; *r* > = 0.5, Large. Significant correlations are shown with bold text

## DISCUSSION

Stroke patients are known to have disability-related low quality of life, but little is known about how the subjective experience of functioning changes within the first year after stroke. We concentrated our study on the subacute rehabilitation period, anticipating only minor improvements in motor and cognitive functioning. The multidisciplinary inpatient rehabilitation unit discharged the patients involved in our study after the Functional Independence Measure (FIM) score showed no improvement in the weekly assessment. As stated in previous studies, we found stroke patients experiencing fewer restrictions compared with their significant others or rehabilitation specialists (17–20). In this study, we observed that patients reported unexpectedly high performances in the ICF core set at 1-month follow-up, compared with rehabilitation team assessment. However, at 12-month follow-up, patients have a tendency to report inferior performance compared with rehabilitation team assessment, while the overall disagreement diminishes. During the 1-month follow-up, a significant discrepancy emerged in the cognitive domains, as patients’ subjective perception of their performance significantly outperformed the team assessment. However, at the 12-month follow-up the overall disagreement diminished. These findings suggest that the perception of functioning among stroke patients undergoes notable changes during the first year, highlighting the dynamic nature of subjective vs objective assessments in post-stroke rehabilitation.

Patients reported no major changes in cognitive and motor performance during the first year after stroke, while team assessment revealed significant improvements in cognitive and motor skills. We suggest that a natural coping process with decreased awareness of impairments at the acute and subacute states can explain the differences between subjective and objective evaluations. Patients in the acute and early subacute phases may have unrealistic expectations of their own performance in daily living, which can lead to a relatively high experience of quality of life in the initial months following stroke. Patients reported major changes in the first 6 months, with improvements in work activity, joining in community activities, and lifting and carrying objects. On the other hand, the team reports significant improvements in motor, ADL, and cognitive skills, while patients experience no change in motor skills or ADL except lifting and carrying objects. Only memory function showed a subjective increase in the cognitive domains from the 1-month to 6-month follow-up. The ICF core set and WHODAS 2.0 subgroups both reported perceived stability in cognitive skills. At 12-month follow-up, patients reported worsening of discomfort and urination function compared with 1-month follow-up. We also found an increase in anxiety from 6- to 12-month follow-up. Further research is necessary to understand this phenomenon, which likely is a reaction on awareness of stroke-caused impairments, and rather reflects the coping process of stroke patients in the first year after diagnosis. The results of our study are similar to those of other studies (16, 19), which found that patients and their caretakers agreed more on the level of functioning at follow-up than at the start of rehabilitation.

Our study reveals that, unlike previous research (16, 19), patients did not perceive significant improvements in motor skills or activities of daily living (ADL) when assessed using the WHODAS 2.0, despite objective evidence of such enhancements. This aligns with Kwon et al. (28), who observed declining trends in WHODAS 2.0 subdomains over the first 3 years post-stroke.

In our rehabilitation approach, we employed the ICF core set for goal-setting and as a dynamic assessment tool throughout various rehabilitation phases. Although patients may not receive direct feedback from team assessments, utilizing the ICF core set as an evaluation and outcome measurement tool can influence patients’ perceptions of their disabilities and limitations, thereby enhancing their focus on rehabilitation outcomes. Systematic use of ICF categories enhances the precision and effectiveness of functional assessments, intervention planning, and interprofessional communication, ultimately improving outcomes for individuals across diverse healthcare settings.

The grading system provides a standardized framework for communicating the extent of a problem, enabling better coordination across healthcare providers and stakeholders and helping to create targeted and effective intervention plans tailored to their specific needs.

Spearman correlation analysis indicated that most ICF subdomains had moderate to substantial correlations with the EQ-5D, a standardized instrument for measuring health-related quality of life (HRQOL). Exceptions included subdomains such as listening, sleeping, breathing, general understanding, speaking, memory, and transferring to the toilet, which did not show significant correlations with EQ-5D. Notably, we observed a shift in these correlations between the 1-month and 12-month follow-ups. Initially, patients regarded cognitive skills as less influential on HRQOL; however, by the 12-month mark, the relationship became more pronounced.

Among the predictors of HRQOL, self-washing and depression emerged as the most significant. Despite the recognized impact of depression on rehabilitation, our study did not find a correlation between depression and motor or ADL skills, even at the 1-year follow-up, whereas other domains exhibited improved interrelations over time.

These findings underscore the complex interplay between subjective perceptions and objective assessments in stroke rehabilitation, highlighting the need for comprehensive evaluation tools that consider both patient-reported outcomes and clinical measurements to effectively address the multifaceted nature of recovery.

Our study also examined the relationship between post-stroke depression and functional recovery, with a focus on motor skills and activities of daily living (ADL). While previous research has identified correlations between depression and motor function, our findings present a nuanced perspective. Yoshida et al. (29) reported a weak negative correlation between depression and motor skills, suggesting that higher motor function is associated with fewer depressive symptoms. However, the longer mean post-stroke evaluation time in their study may influence these results. Similarly, Tur et al. (30) found that post-stroke depression contributes to lower Functional Independence Measure (FIM) scores in the subacute rehabilitation phase.

In contrast, our analysis did not reveal a significant correlation between WHODAS 2.0 motor and ADL functions and ICF depression in outpatient rehabilitation settings.

Post-stroke depression is often persistent, and cognitive decline after stroke can impact mood and emotions (31). Our findings align with previous studies, showing no correlation between patient-reported emotional functions and cognition at initial assessment, but a moderate correlation at 1-year follow-up, despite objective improvements in cognitive functions during the first year.

It is important to note that our study is a single-centre analysis with a population selected for optimal rehabilitation potential, which may limit the generalizability of the results. Additionally, the retrospective design may introduce reporting bias.

In conclusion, the first-year rehabilitation outcomes of stroke patients reveal significant differences in functioning between self-assessments by stroke survivors and evaluations by the rehabilitation team. Notably, the overall disagreement diminished at the 12-month follow-up. Cognitive domains exhibited the greatest inter-observer differences, with patients perceiving restrictions as less severe.

Objective assessments indicated significant improvements in cognitive, motor, and ADL skills, while patients reported greater enhancements in work activity, household tasks, community participation, lifting and carrying objects, and memory functions.

The strongest predictors of quality of life, as measured by the EQ-5D, were self-washing and depression. Cognitive functions did not correlate with EQ-5D at initial assessment, but a moderate correlation emerged at the 12-month follow-up. Over the 1-year period, the overall correlation between WHODAS 2.0 subdomains improved. No correlations were found between depression and mobility or self-care.

The ICF core set, encompassing both objective and subjective assessments from patients and rehabilitation teams, proves to be a valuable instrument for measuring rehabilitation outcomes.

These insights underscore the importance of integrating both patient-reported outcomes and clinical evaluations to effectively address the multifaceted nature of stroke recovery.
